# Knowledge, stigma, and beliefs toward mental illnesses among schoolteachers in Damascus

**DOI:** 10.1177/00207640211015701

**Published:** 2021-05-09

**Authors:** Youssef Latifeh, MHD Wael Jaredh, Lulia Nasri, Duaa Shriedy, Ayat Al-Mahdi, MHD Wasim Murtada

**Affiliations:** 1 Syrian Private University, Syria; 2 Faculty of Medicine, Damascus University, Damascus, Syria, Syrian Arab Republic

**Keywords:** Stigma, mental illnesses, schoolteacher, Syria

## Abstract

**Background:**

Teachers have an important role in promoting the mental wellbeing of their students, hence their knowledge and attitudes toward mental health disorders should be assessed. A very few studies regarding this topic were conducted in Syria, but due to the recent events which have had occurred the country, it is essential to deal with students who suffer from mental illnesses professionally especially that such disorders may be stigmatized by the society.

**Aims:**

This paper aims to investigate knowledge, beliefs, and attitudes toward mental disorders in a sample of Syrian schoolteachers.

**Method:**

A cross-sectional study using self-administered questionnaire was conducted in Damascus and Refdimashq, involving 400 teachers from 16 schools.

**Results:**

The results showed that the prevalence of stigmatizing positions toward psychiatric illnesses was low among teachers. The contributors did not state a correlation between spiritual beliefs and psychological disorders. It was also found that teachers were neutral in their knowledge about mental illnesses and psychiatric treatment or interventions along with their mental health resources. However, 42.5% of the participants use the internet for such purposes.

**Conclusion:**

In general, teachers of Damascus and its Refdimashq had a reasonable degree of awareness about mental disorders and treatments. Furthermore, neither stigma nor the relation between religious thoughts and mental disorders had been ascertained.

## Introduction

Mental health plays a vital role in the wellbeing of humans. Healthy individual function properly in a society. Psychiatry is the branch of medicine which directly contributes to the maintaining of mental health and its attributes, according to the American psychiatry association. A study was conducted in Egypt mentioned that 5,000 years ago there was no distinction between psychiatric and physiological disorders consequently stigmatizing attitudes were not existent (Okasha, 2004). However, nowadays mentally ill patients face social discrimination due to a widespread stigma resulting in low self-esteem and social isolation (Corrigan, 1998). Other studies pointed out that being a symptomatic psychiatric patient hindered the possibility of being hired by employers or expressing oneself freely in public (Corrigan et al., 2001). Thereafter, the overwhelming stigma inhibited those patients from seeking psychiatric assistance or evaluation. Further studies suggested that the majority of the public in the United States (Link, 1987; Phelan et al., 2000; Roman & Floyd, 1981) and in many Western European nations showed stigma toward mentally ill people (Bhugra, 1989; Madianos et al., 1987). Recent studies indicated that roughly half of psychiatric disorders appear by mid-adolescence (Kessler et al., 2007), making schools have an essential role in providing guidance to their students and educating and raising awareness of the public (Imran et al., 2018). Studies have found that 10% of adolescents sought guidance from their teachers when faced with problems (Oban, 2011). Therefore, schools have become a consulting centers where students could get help safely (Kütük et al., 2016). Furthermore, it is estimated that 22% of people living in conflict regions have psychiatric disorders (Charlson et al., 2019). Since the beginning of the Syrian civil war, drastic changes affected the normal everyday lifestyle, education, research, and awareness campaigns toward mental health. Besides, even before the war, mental health care in Syria was transparently undersupply; to put it simply, there are only 70 psychiatrists and two public psychiatric hospitals for 21 million people (WHO, 2013). According to a study that conducted on Syrian students, over half of the sample had at least one psychological disorder. The dominate one was PTSD, followed by depression and anxiety (Perkins et al., 2018). Psychological counseling in Syria is limited by school psychologists graduated from faculty of education. Syria is one of the worst areas that have conflict for (McIntyre, 2020). By detecting other regional wars, the death rate in Syria was more than the others (McIntyre, 2020). The consequences of this war have negatively impacted the poorly developed research environment in Syria. However, to our best knowledge, this is the first study in Syria to evaluate the teachers’ knowledge and attitudes toward mental disorders, treatment, and intervention methods. This study also aims to assess the mental health resources teachers have and examines their perspective about the linkage between spirituality, religion, and mental illnesses.

## Methods and materials

### Study design, sample, and procedures

This cross-sectional study was conducted between September of 2019 and March of 2020. According to statistics in 2019, there were 583 schools and 17,692 teachers in Damascus and Refdimashq (the two largest provinces in Syria). Syria has public and private schools, which have national mandatory curriculum. Education is compulsory until the end of the middle school. Teachers are usually graduated from Syrian Universities, graduated teachers can still get training diploma which is not obligatory. Our sample consisted of 400 teachers out of a total of 17,692 teachers in the two provinces, that is, 2.2% which were chosen from 16 schools from the urban areas of Damascus and Refdimashq, based on a statistical algorithm which used the standard deviation and error rate. The schools were randomly chosen after implementing the random number tables. We obtained an informed consent from all participants, and ethical review was approved by the faculty of medicine at Damascus University. The questionnaires then were randomly distributed to teachers, based on a random sampling method. A research assistant was always present in the classroom to answer the teacher’s queries. The questionnaire required less than 20 minutes to be completed. After an eligibility criterion was established, teachers were assigned identification numbers and randomized using online software, Research Randomizer (www.randomizer.org). The participants included the entire educational staff. Participants were excluded if they had less than 1 year of experience, also uncompleted questionnaires were ruled out.

### Tools

We collected socio-economic and demographic data using a questionnaire that included (gender, age, marital status, date of birth, home-town, residence, income, teaching experience, teaching subject, cigarette smoking, and family history of psychiatric disorders). We used another questionnaire from a research published in 2010 by Latifeh (2010). Which was designed using globally standardized stigma studies to evaluate the awareness, stigma, and attitude toward mental illness (Fung et al., 2007; King et al., 2007). The validity of that survey was assessed in the above mentioned research. This questionnaire included 36 self-evaluation queries, each query was rated on a 5-point liker (5PL) scale (liker scales) ranging from strongly disagree to strongly agree.

### Statistical analysis

We analyzed the data obtained using SPSS™ version 20 and used frequency and percentage to represent categorical data such as socio-demographic variables. Bivariate analysis using Chi-square test was done to determine the connection between the demographic variables and the main concepts of our study and the internal relation among the concepts. Statistical significance was set at *p*-value of less than or equal to .05.

## Results

Table 1 represents the main demographic details. This study was done on 418 schoolteachers, but 18 forms were excluded due to missing data or the short period of teaching experience (less than 1 year). Therefore, the response rate was 95.7%. In addition to the demographic information that is shown in the table below, we found that 32.1% were primary teachers and counseling psychologists and administrators, 8.2% were teaching math, 9.7% were teaching physics, science, and chemistry while 50% were teaching literature, languages, and sports. Meanwhile, most of them 90.8% were living in Damascus and Refdimashq. Whereas 74.3% were originally from Damascus or Refdimashq, 11.7%, 7.3%, 6.7%, came from northern, southern, and western areas of Syria respectively.

**Table 1.** Demographic characteristics of participants (*N* = 400).

Characteristics	*N* (%)	Std.	Std. err	Variance
Gender		0.464	0.023	0.215
Female	288 (71.8%)			
Male	112 (28%)			
Age		0.971	0.049	0.943
20–30 year	88 (22%)			
31–40 year	149 (37.3%)			
41–50 year	107 (26.8%)			
>50 year	56 (14%)			
Marital status		0.551	0.028	0.303
Single	132 (33%)			
Married	250 (62.5%)			
Other	17 (4.3%)			
Cigarette smoking				
Current	80 (20%)			
Not current	320 (80%)			
Residence		0.290	0.015	0.084
Urban	363 (90.8%)			
Rural	37 (9.3%)			
Personal income		0.742	0.037	0.551
Sufficient	153 (38.3%)			
Intermediate	188 (47%)			
Not sufficient	59 (14.8%)			
Working experience		9.371	0.469	87.811
Less 5 year	103 (25.8%)			
6–10 year	97 (24.3%)			
11–20 year	132 (33%)			
21–30 year	54 (13.5%)			
More 30 year	14 (3.5%)			
Family history of psychiatric disorder		0.366	0.018	0.134
Yes	51 (12.8%)			
No	347 (86.8%)			

In this section, we will discuss five concepts to explore teachers’ views on psychiatric and mental illnesses and the relation with the demographics.

### Spiritual and religious possible issues

We asked the participants six questions related to wrongly interpreted and generally accepted ideas originated from religious teachings (Table 2). The weighted average of this relation was 3.85 which lay in 3.40 to 4.20 interval and with 0.679 Std. which indicated that such ideas highly likely could act as a contributing cause to mental illnesses. Analytically, the obtained average was positioned in the high level area of the scale [3.40–5] (Timur & Tasar, 2011). In order to further analyze the relation within the demographic data and the first concept, a chi-square test of independence with *p*-value = .05 for significance was performed. The results of the test suggested a strong relation between some demographic variables and the first concept (gender, marital status, cigarette smoking, and residence) with *p*-value (<.001, <.001, <.001, and .023, respectively) whereas other variables did not have any significant relations with the first concept (Table 5).

**Table 2.** Spiritual and religious issues.

Questions		Strongly agree	Agree	Neutrally	Disagree	Strongly disagree	Mean	Std. deviation	Rank
Believing in supernatural beings and witchcraft causes mental illnesses.	*N*	46	98	35	73	148			
	%	11.5	24.5	8.8	18.3	37	3.45	1.474	5
Believers and religious people won’t have mental illnesses.	*N*	100	70	59	80	91			
	%	25	17.5	14.8	20	22.8	2.98	1.513	6
Psychiatry cannot diagnose or treat the psyche, since it is unperceivable	*N*	33	66	66	103	132			
	%	8.3	16.5	16.5	25.8	33	3.59	1.316	4
Reaching out for psychotherapy is translated to lack of religious faith.	*N*	5	16	23	51	305			
	%	1.3	4	5.8	12.8	76.3	4.59	0.866	1
Using psychotropic medications is related to weak personality and lack of religious faith	*N*	18	14	32	65	271			
	%	4.5	3.5	8	16.3	67.8	4.39	1.073	2
There is a contradiction between religion and psychiatry.	*N*	19	30	61	66	224			
	%	4.8	7.5	15.3	16.5	56	4.12	1.196	3
Total	Std. deviation 0.679			Weighted mean 3.85					

### Teachers’ awareness of the mental illness

We examined the teachers’ awareness of mental disorders using 11 questions (Table 3). The results revealed that the second concept was neutral in regard to mental illnesses. According to (5PL) scale the average existed in the interval 2.60 to 3.39 which is considered a moderate level. However, according to (Table 5), there was a strong relationship between the second concept and the variable ‘Age of instructor’ with *p*-value .037, whereas the other variables did not have any significant relationship (Table 5).

**Table 3.** Teachers’ awareness of the mental illness.

Questions		Strongly agree	Agree	Neutrally	Disagree	Strongly disagree	Mean	Std. deviation	Rank
Bad parenthood and physical abuse are the main reasons of mental illness.	*N*	172	180	5	33	10			
	%	43	45	1.3	8.3	2.5	1.82	0.984	11
Reporting badly behaved students to psychotherapy is advantageous.	*N*	107	181	25	40	47			
	%	26.8	45.3	6.3	10	11.8	3.65	1.293	6
Seeking psychotherapy is important for myself when needed.	*N*	175	116	62	22	22			
	%	43.8	29	15.5	5.5	6.3	3.99	1.174	5
Psychiatrists should be very friendly and open with their patients.	*N*	195	99	44	33	29			
	%	48.8	24.8	11	8.3	7.2	2.01	1.259	10
Psychiatrists are capable of analyzing our thoughts; extreme caution should be taken upon visiting them.	*N*	40	111	60	61	128			
	%	10	27.8	15	15.3	32	3.32	1.42	8
Mental illnesses are harder to deal with than physical or physiological illnesses.	*N*	56	55	101	67	120			
	%	14	13.8	25.3	16.8	30.0	3.33	1.457	7
Schizophrenic patients suffer from a split in their personality or have two personalities.	*N*	148	124	102	11	15			
	%	37	31	25.5	2.8	3.8	2.05	1.036	9
Mental illnesses cannot be cured.	*N*	9	15	40	75	261			
	%	2.3	3.8	10	18.8	65.3	4.41	0.969	2
Psychoanalysis does not have a firm scientific basis.	*N*	23	45	36	88	208			
	%	5.8	11.3	9	22	52	4.03	1.257	4
Having mentally ill students is not a major issue.	*N*	11	13	24	58	294			
	%	2.8	3.3	6	14.5	73.5	4.53	0.947	1
Reporting students who show signs of mental illness to their parents are important.	*N*	218	123	25	18	15			
	%	54.5	30.8	6.3	4.5	3.8	4.31	1.156	3
Total	Weighted mean 3.06				Std. deviation 0.444				

### Stigma toward mental disorders

The participants then were asked 10 questions in order to examine their perspective on the stigma associated with psychiatric disorders (Table 4). The weighted average was 3.77 with std. 0.531. According to (5PL) scale the average existed in the interval which is considered a high level. Moreover, the findings informed us that there was no significant relationship between the third concept and any of the demographical variables (Table 5).

**Table 4.** Stigma toward mental disorders.

Questions		Strongly agree	Agree	Neutrally	Disagree	Strongly disagree	Mean	Std. deviation	Rank
Psychiatry treats insanity, madness, and dementia.	*N*	53	117	68	62	100			
	%	13.3	29.3	17.0	15.5	25.0	3.10	1.405	8
Thinking about psychotherapy and psychiatry makes me anxious.	*N*	18	38	16	78	250			
	%	4.5	9.5	4.0	19.5	62.5	4.26	1.177	4
Suffering from a mental illness myself is highly unlikely.	*N*	44	52	147	73	84			
	%	11.1	13.0	36.8	18.3	21.0	3.23	1.377	7
Visiting a psychiatrist induces feelings of shame and guilt.	*N*	18	50	28	75	229			
	%	4.5	12.5	7.0	18.8	57.3	4.12	1.240	5
Seeking help from a psychiatrist is a sign of madness.	*N*	45	119	86	57	93			
	%	11.3	29.8	21.5	14.2	23.3	3.09	1.348	9
Suicide is much easier than visiting a psychiatrist when suffering from a mental illness.	*N*	3	5	14	10	368			
	%	0.8	1.3	3.5	2.5	92.0	4.84	0.610	1
Almost everyone is fearful from seeking the help of psychiatrists.	*N*	157	134	45	34	29			
	%	39.3	33.5	11.3	8.5	7.2	2.08	1.321	10
Being considered a psychiatrist patient is fearful.	N	15	17	18	38	312			
	%	3.8	4.3	4.5	9.5	78.0	4.54	1.023	2
Mentally ill individuals should be treated in mental facilities since they are aggressive.	*N*	6	38	55	127	174			
	%	1.5	9.5	13.8	31.8	43.5	4.06	1.042	6
Psychiatric disorders are infectious.	*N*	15	32	35	46	272			
	%	3.8	8.0	8.8	11.5	68.0	4.32	1.151	3
Total	Weighted mean 3.77				Std. deviation 0.531				

**Table 5.** Correlation between demographic variables and main concepts.

Independent variable(s)	Wrong concepts and their relation to the spiritual and religious issues		Awareness toward mental illnesses		Stigma toward psychiatric disorders		Psychiatric interventions and treatment		Information resource	
	χ^2^	*p-*Value	χ^2^	*p-*Value	χ^2^	*p-*Value	χ^2^	*p-*Value	χ^2^	*p-*Value
Gender	88.559	<.001	5.629	.689	52.015	.626	27.679	.957	17.358	.743
Age	48.221	.863	22.007	.037	65.684	.930	67.994	.311	35.391	.356
Marital status	110.793	<.001	18.697	.096	96.505	.166	43.344	.972	29.679	.633
Cigarette smoking	104.810	<.001	12.919	.115	51.062	.662	31.926	.870	22.406	.436
Residence	34.433	.023	2.125	.713	35.388	.159	48.098	.001	12.345	.338
Personal income	69.117	.197	13.023	.367	74.105	.771	53.680	.729	60.386	.003
Working experience	65.394	.881	12.818	.686	95.316	.871	89.059	.332	44.239	.462
Family history of psychiatric disorder	42.572	.361	5.744	.676	45.258	.847	31.951	.869	11.456	.968
1st concept	–	–	668.138	<.001	1,157.974	<.001	515.526	.001	227.645	.348
2nd concept	668.138	<.001	–	–	885.080	<.001	677.245	<.001	298.329	.026
3rd concept	1,157.974	<.001	885.080	<.001	–	–	718.870	<.001	299.712	.622
4th concept	515.526	.001	677.245	<.001	718.870	<.001	–	–	233.194	.447
5th concept	233.194	.447	298.329	.026	299.712	.622	233.194	.447	–	–

### Knowledge of psychiatric treatment and interventions

Knowledge of psychiatric treatment and interventions was the fourth analyzed concept. The findings indicated that one in four teachers agreed that psychotropic medications lower the intellectual abilities of patients, including 6.3%, 18.3% who strongly agreed and agreed respectively. The calculated mean was 3.45 with *SD* of 1.259. Also, 26.1% of teachers agreed that psychotropic medications may lead to have suicidal thoughts, including 9.3% who strongly agreed with mean of 3.28 and *SD* of 1.263. Almost one-third of participants agreed that they can control medication dosage since they know their own condition better than experts, including 11.8% who strongly agreed and 21% agreed with mean 3.10 and *SD* of 1.291. Whereas 16.5% strongly agreed and 20.8% agreed that psychiatric medications lead to addiction with mean of 2.85 and *SD* of 1.215. Meanwhile, more than half of the participants strongly agreed 22.5% and agreed 36.3% that psychiatric therapy sessions are mainly dialogue sessions with mean of 2.67 and *SD* of 1.402. Roughly, the majority agreed that psychotherapy sessions are the most important and effective treatment for patients with mean of 2.67 and *SD* of 1.402. The weighted average for these questions was 2.89 with *SD* of 0.721. The results pointed out that the fourth concept was generally neutral. According to the 5PL scale, them mean was found in the interval 2.60 to 3.39 which is considered a moderate level. The results provided a significant relationship between this concept and the variable ‘residency’ with *p*-value 0.001, whereas there were no other relations (Table 5).

### Resources of psychiatric knowledge

The final discussed concept was mental illnesses resources. The teachers answered three questions about their source of information in regard to mental illnesses. Interestingly, three-quarters of participants, 11.5% strongly agreed and 39% agreed, used academic medical books and journals as trusted sources of information about mental illnesses with a mean of 3.78 and *SD* of 1.218. About 42.5% of teachers depended on social media and the internet for getting information, including 7.5% who strongly agree with a mean of 3.18 and *SD* of 1.296. However, half of the teachers 50.3% agreed to go back to TV, newspapers, and radio, including 45% who strongly agreed with a mean of 2.93 and *SD* of 1.300. The cumulative mean of these finding was 3.29 and 0.761 *SD*, which indicated that the result of these questions is generally neutral. According to the (5PL) scale the mean is found in the interval 2.60 to 3.39 which is considered a moderate level. The fifth concept had a strong relation between the variable ‘personal income’ with *p*-value .003 (Table 5). A chi-square test was conducted to explore whether there is any relationship among the concepts themselves? The results provided significant relations within all the concepts. However, the fifth concept was only related to the second concept at a *p*-value of .026 as (Table 5) outlines.

## Discussion

Teachers are the main participant in preparing new generations (Reddy, 2018). Thus, having teachers who are aware of the impact of mental health on the students is essential (Alharbi et al., 2019; Gur et al., 2012; Parikh et al., 2016). However, it is equally important to consider a teacher’s mental health as it is a major contributing factor to the classroom positive outcome (Roberts et al., 2016; Sandilos et al., 2015). To the best of our knowledge, this is the first study that assessed teacher’s knowledge and attitude toward mental illness among a Syrian sample.

### The spiritual and religious issues

Our data revealed that the majority of teachers did not refer to a relation between spiritual beliefs and mental illnesses. Similarly, a Pakistani study could not prove whether Islam teachings reduce stigma toward mental illnesses (Javed et al., 2006). Other studies demonstrated that extreme levels of religious attachment could potentially provide a foundation that mentally ill individuals could hold on to (Lukachko et al., 2015). In contrast, studies conducted in east Asia pointed out that supernatural powers cause psychiatric disorders, and traditional healer ought to be sought (Lauber & Rössler, 2007b). Our data indicated that many teachers strongly disagreed with the role of supernatural powers and witchcraft to cause mental illness, going to a psychiatric clinic or using psychotropic medications is an indication of lack of religious faith, the existence of a conflict between psychiatric and faith. Perhaps this is due to the many TV programs that have fought against the themes of witchcraft and sorcery since the 1990s. The majority of teachers strongly agreed that believers and religious people will not have mental illnesses. It’s possible that they are setting the religiously observant man as a role model. Our results provided a strong correlation between the first concept and variables such as gender, marital status, being a smoker, and residence. This is in concordance with a previous study that showed an association between residence and knowledge of mental disorders, in which it was found that urban people were more likely to consider mental illnesses as God’s punishment (Kishore et al., 2011).

### Awareness of mental illnesses

In terms of awareness, we did not find any differences with respect to gender, unlike a different study where it was found that women have a better understanding concerning mental health (Mann & Himelein, 2004). We also noticed a significant relationship between age and awareness. Our study revealed that teachers were neutral in that their answers varied based on the question. To illustrate, 84.1% refused that mental illnesses did not have a cure. Furthermore, 72% accepted to see a psychiatrist when necessary, consistently with a Turkish study where school counselors visited a psychiatrist more than the remaining population (Gur et al., 2012). Additionally, the majority of our teachers assumed bad parenting as the cause of mental disorders, in compliance with our results, the information related to mental illnesses was not enough according to a published Pakistani paper (Lauber & Rössler, 2007a). Also, there was insufficient information in terms of evidence-based practice (Reinke et al., 2011). However, this study showed that over 50% of participant have insufficient knowledge about schizophrenia. Such a finding is in accordance with the findings presented in a different study which showed that teacher stigmatized mental illnesses, particularly schizophrenia (Lauber & Rössler, 2007a). Another study revealed that the stigma was more noticeable toward schizophrenia than depression (Mann & Himelein, 2004). A possible explanation for our results may be due to the fact that many Syrian teachers undergo programs that provide useful yet inadequate psychiatric principals. Therefore, establishing stronger mandatory programs to help teachers better understand mental illnesses is vital.

### Stigma toward psychiatric disorders

The taken attitude in respect of mental illnesses plays a serious role in affecting the mental health of those who suffer from mental illnesses. The stigma has a noticeable impact on the demeanor as well as the appropriate medication (Gur et al., 2012). Therefore, it was important to assess teachers’ attitude toward mental illnesses. The results showed that teachers did not stigmatize psychiatric disorders. This finding was opposing the findings of a Turkish study where the preponderance of teachers held a negative attitude toward mental disorders (Gur et al., 2012). Moreover, a study was done by Mann et al. showed that counseling teachers adopted an affirmative attitude despite their negative thoughts (Mann & Himelein, 2004). Other Indian study reported that three-quarters of school teachers had a stigma to a depressive case vignette (Venkataraman et al., 2019). A potential explanation to such findings in the study is the raise of the Syrian civil war which induced various mental disorders and shifted or showed clearly the attitude of the public toward mental disorders. Simultaneously, the ease of access to trusted and accurate information on the internet in regard to mental disorders could play an important role in destigmatize mental illnesses. In accordance with a different study, our data did not find any relationship between demographic variables and the attitude taken toward mental illnesses (Gur et al., 2012). Whereas a Nigerian study pointed out that attitude may be affected by work experience, academic background, and gender (Aghukwa, 2009).

### Knowledge of psychiatric interventions and information resources about mental illnesses

In terms of means of interventions, the psychotherapy sessions were an effective method seen by a number of our participants. Many teachers did not know whether a relation between psychotropic medications and addiction with suicide exist. Furthermore, 50% of teachers strongly disagreed with the notion that psychotropic medication negatively affect intellectual abilities, contrasting a Colombian study which showed opposing results (Padilla et al., 2018). Additionally, the majority of teachers believe psychotropic medications to be disadvantageous (Jorm, 2000). Nevertheless, more than half of an Australian sample preferred positive social support over medications as treatment for depression (Kelly et al., 2006). The participant of this study had a neutral opinion regarding psychiatric interventions and treatment, and this was anticipated as even medical students had a lack of information about psychiatric drugs, especially in developed countries like India according to a study (Chawla et al., 2012). Our analyses yielded a significant relationship between the following variables; residence and personal income and the fifth concept. Whereas a Norwegian study showed that gender was highly correlated with the fifth concept (Ghanizadeh et al., 2006). We also found that some teachers relay on TV, newspaper, internet as psychiatric information sources while TV, relatives, friends, periodicals, and magazines were depended on in other studies (Hamre et al., 2009). An American study showed that high-income socioeconomic and health care workers depended on medical universities and federal web sites as a trusted resource for information (Dutta-Bergman, 2003). The results revealed that the stigma was related to teachers’ spiritual beliefs, awareness, and knowledge toward mental illnesses and treatment interventions. While an Indian study showed that religiousness was an essential part of human life and had a certain relation with normal mental health (Behere et al., 2013). In addition, there were relations between all concepts of our research except the information sources which was only related to the awareness toward mental illnesses. This is in concordance with an Indian study which revealed that media is a big influencer device on people’s opinions (Srivastava et al., 2018).

## Strengths and limitations

Despite the importance of studying the prevalence of stigma toward Psychiatric issues, few studies have reviewed the stigma prevalence in the area. Therefore, this is the first description of the level of teachers’ stigma in the Syrian population. We also demonstrated the relation between its concepts which was not done before in similar studies. Additionally, the sampling method was randomized making the bias minimized. Despite these strengths, this study should be interpreted with consideration of some limitations. Firstly, it is difficult to confirm that our sample was representative of the population of teachers of Damascus and Refdimashq since we did not include all school types Additionally, some teachers include in our study were teachers who were who were presently teaching at Damascus and Refdimashq schools but were conditionally transferred from their original schools in other governorates because of the Syrian war. Moreover, the survey did not go into detail about the academic qualifications of teachers. Finally, more studies with larger samples are still needed to fully understand the factors that affect teachers’ attitudes.

## Conclusion and recommendations

In conclusion, teachers’ stigma toward mental illness presents a major public health concern worldwide due to its negative impact on students. Our study found that the majority of teachers in Damascus and Refdimashq schools had a moderate level of awareness about mental disorders and psychiatric treatments. Moreover, we found no correlation between religious beliefs and psychiatric disorders. It is important to state that the Syrian environment has not been stable for a long period due to the political conflict and lack of security. It is expected that these circumstances would have a negative effect on physical and mental health, so any intervention that targets the Syrian population should be appropriate to the environment and respectful to the barriers of the country. In spite of the fact that numerous attempts have been done to alleviate the Psychiatric impacts of the Syrian crisis (Syrian Arab Republic Annual Report, 2015; WHO, 2014), more effort is still required. Anti-stigma campaigns should be done for the future progress of mental health.
